# Antagonism of the Neonatal Fc Receptor as an Emerging Treatment for Myasthenia Gravis

**DOI:** 10.3389/fimmu.2019.03052

**Published:** 2020-01-10

**Authors:** Karissa L. Gable, Jeffrey T. Guptill

**Affiliations:** ^1^Department of Neurology, Duke University School of Medicine, Durham, NC, United States; ^2^Duke Clinical Research Institute, Durham, NC, United States

**Keywords:** FcRn antibodies, myasthenia (myasthenia gravis–MG), novel therapeutic, IgG, autoimmune, treatment, clinical trial review

## Abstract

Myasthenia gravis is an autoimmune disease in which immunoglobulin G (IgG) autoantibodies are formed against the nicotinic acetylcholine receptor (AChR) or other components of the neuromuscular junction. Though effective treatments are currently available, many commonly used therapies have important limitations and alternative therapeutic options are needed for patients. A novel treatment approach currently in clinical trials for myasthenia gravis targets the neonatal Fc receptor (FcRn). This receptor plays a central role in prolonging the half–life of IgG molecules. The primary function of FcRn is salvage of IgG and albumin from lysosomal degradation through the recycling and transcytosis of IgG within cells. Antagonism of this receptor causes IgG catabolism, resulting in reduced overall IgG and pathogenic autoantibody levels. This treatment approach is particularly intriguing as it does not result in widespread immune suppression, in contrast to many therapies in routine clinical use. Experience with plasma exchange and emerging phase 2 clinical trial data of FcRn antagonists provide proof of concept for IgG lowering in myasthenia gravis. Here we review the IgG lifecycle and the relevance of IgG lowering to myasthenia gravis treatment and summarize the available data on FcRn targeted therapeutics in clinical trials for myasthenia gravis.

Myasthenia gravis (MG) is an autoimmune disease affecting the neuromuscular junction that manifests in clinical symptoms, such as dyspnea, dysphagia, diplopia, dysarthria, ptosis, and fatigable muscle weakness. Symptoms often fluctuate in severity, are generally fatigable, and improve with rest. It is estimated that this disorder affects ~60,000 people in the United States (1). Patients with mild disease experience ocular symptoms of diplopia and intermittent ptosis and, on the other end of the spectrum, patients with severe disease experience generalized weakness that can progress into myasthenic crisis resulting in respiratory insufficiency and need for ventilatory support.

Neuromuscular junction function involves acetylcholine release from the motor nerve, binding of acetylcholine to the acetylcholine receptor (AchR) on the post-synaptic membrane, followed by generation of muscle end plate potentials. Once the end plate potential reaches threshold an action potential is generated, resulting in normal muscle contraction. The etiology of the autoimmune process in myasthenia gravis is unclear in most cases, however, the autoantibodies generated in the disease target the nicotinic AchR or other components of the post-synaptic neuromuscular junction. Interference with downstream signaling at the post-synaptic junction reduces the ability of the end plate potential to reach the threshold needed to trigger an action potential, ultimately resulting in the clinical manifestation of fatigable or persistent muscle weakness (2).

Current pharmacologic approaches to treat MG either try to control the symptoms (e.g., cholinesterase inhibitors) or suppress or modulate the immune system. Corticosteroids, steroid–sparing agents, therapeutic plasma exchange (TPE), and immunoglobulin infusions (IVIg) are currently the most common treatment modalities. However, each of these treatments can be associated with various side effects. Corticosteroid treatment, especially at high doses over the long term, is associated with a myriad of potential complications, such as steroid-induced diabetes, bone density loss, accelerated cataract formation, gastrointestinal ulcer formation, hypertension, and peripheral edema. Commonly used steroid-sparing agents in the United States, such as azathioprine or mycophenolate mofetil, suppress the immune system and increase risk of infection as well as slightly increase the incidence of certain cancers, such as squamous cell cancer and lymphoma, respectively.

IVIg infusions, TPE, and in some countries, immunoadsorption are used in the setting of myasthenic exacerbations and crisis. Immunoglobulin infusions do not widely suppress the immune system but rather modulate the autoimmune response to minimize the effect of the autoantibodies directed against the post-synaptic receptors. IVIg is associated with rare, but severe adverse events, such as thrombosis, aseptic meningitis, and allergic reactions. In fact, it has been reported that though adverse systemic reactions are rare with subcutaneous IG infusions, they are relatively common with IVIg infusions, occurring in 20–50% of patients and 5–15% of all IVIg infusions (3). There can also be supply issues given that IVIg is a blood product and requires healthy donors. In many cases it is also not a viable long term treatment given relatively common issues with intravenous access needed for the infusions or the need for a long-term indwelling line; subcutaneous administration may help alleviate those issues.

TPE effectively lowers circulating IgG blood levels, including pathogenic autoantibodies, and provides proof of concept for the clinical effectiveness of lowering IgG levels to treat MG (4, 5). One plasma volume exchange reduces serum immunoglobulin levels by 60% and total body immunoglobulin stores by 20% (6). Five sessions of TPE can lower IgG and autoantibody levels by ~75–80%. Recovery of IgG levels close to a baseline level occurs ~6 weeks after a course of TPE (5). TPE has limitations as a maintenance treatment given intravenous access limitations and many centers perform the procedure using a central line. Also, there is also the potential for intolerance due to allergic reactions to the solutions used in the exchange procedure, blood pressure fluctuations or hematologic side effects. TPE removes coagulation factors and complement proteins, requires colloid replacement, most often with albumin replacement, and requires special equipment and expertise (7). Due to the depletion of coagulation factors, treatment is often performed every other day to allow for natural recovery of these clotting factors. On occasion, during the course of TPE treatment, infusion with fresh frozen plasma is required to replace these clotting factors if they are significantly depleted. The required spacing of TPE adds to the time consuming nature of this type of treatment. Given the limitations and risks of the currently available treatments of myasthenia gravis, there is need for treatments that could provide benefits similar to TPE or IVIg and that do not suppress the immune system.

## The Neonatal Fc Receptor as a Therapeutic Target

**Figure 1:**
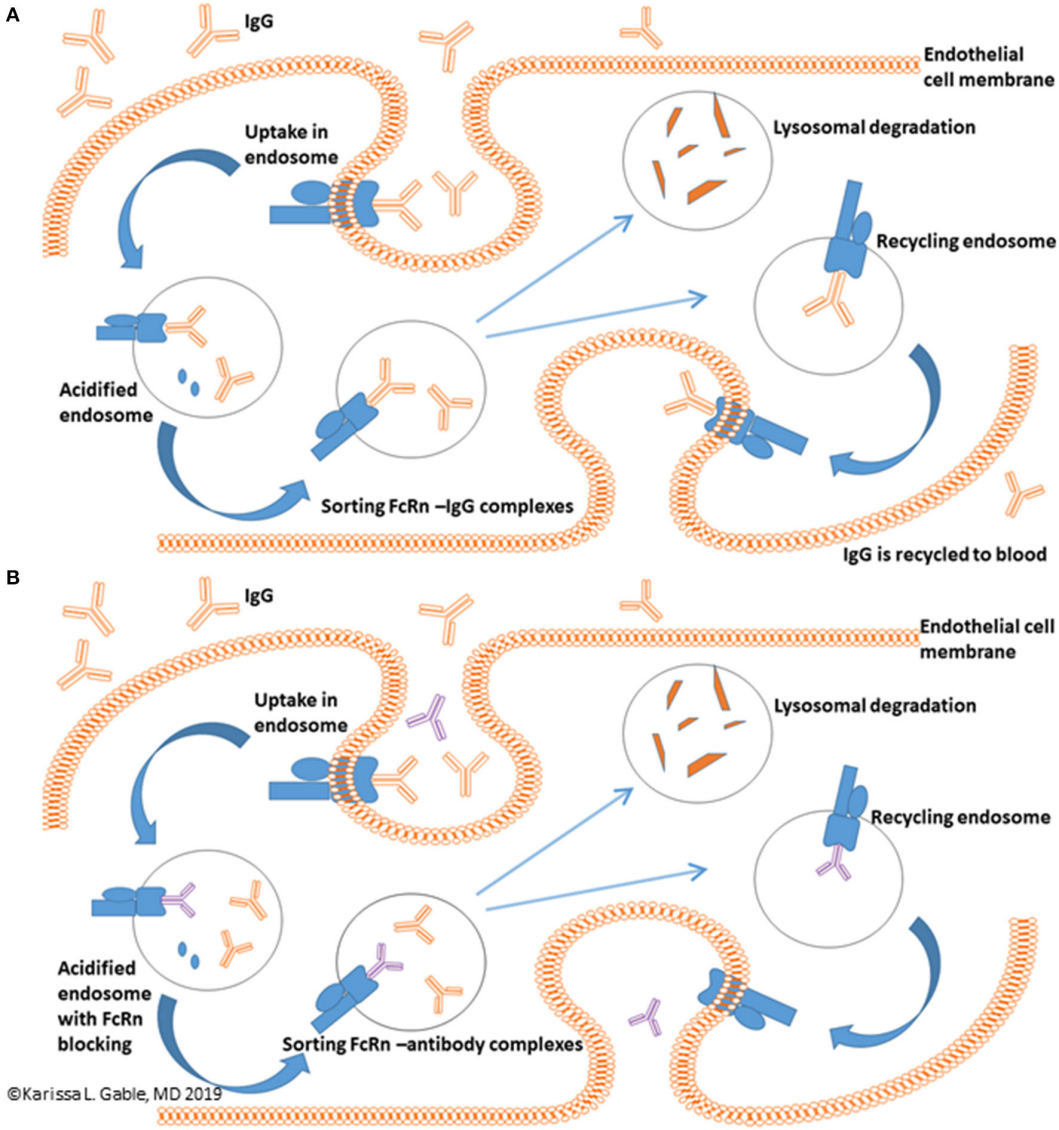
FcRn mediated recycling of IgG. **(A)** IgG recycling begins with IgG binding to the FcRn with IgG uptake into an endothelial cell via an acidified endosome with ultimate release of IgG back into the blood. **(B)** Binding of anti-FcRn therapeutic to the FcRn receptor leaves unbound IgG in the endosome which undergoes lysosomal degradation and reduces circulating IgG levels. Blue receptor, FcRn protein; Blue oval, albumin; Orange, IgG; Purple, anti-FcRn therapeutic.

A novel potential treatment approach is targeting the neonatal Fc Receptor (FcRn). FcRn mediated IgG recycling accounts for passive short-term humoral immunity that is provided *in utero* from mother to offspring (8). In adults, FcRn is expressed in muscle, skin, and vascular endothelium and is critically important to the life cycle of IgG (9–12). In summary, the normal mechanism of directional transport and recycling involves IgG binding to FcRn on the surface of an endothelial cell. This is followed by passive pinocytosis of IgG bound to FcRn into the cell via an acidified endosome. Unbound protein is relegated to lysosomal degradation, whereas IgG bound to FcRn is transcytosed and released back into the serum at physiologic pH. IgG and albumin make up 90% of the serum protein content and the FcRn-mediated recycling process extends the serum half-life of both proteins and is responsible for the 21 days half-life of IgG (Figure 1A) (8, 13, 14). It has been estimated that the FcRn-mediated IgG recycling rate is 42% greater than the rate of IgG production, indicating that recycling of IgG, not its production, is the dominant process for maintaining IgG plasma concentrations in humans (14). Thus, FcRn serves a vital function in maintaining serum IgG levels. Other immunoglobulins, such as IgM, are not involved in FcRn mediated recycling. Inhibiting FcRn recycling is overall expected to be a promising therapeutic target for lowering all IgG subclasses, including IgG4, which has unique properties, such as the ability of IgG half-molecules to recombine randomly with other half-molecules via Fab arm exchange (15).

If binding of IgG to FcRn is inhibited, the expected effect is enhanced IgG catabolism and a reduction in serum IgG concentrations, an effect similar to TPE (Figure 1B). This mechanism of action is potentially quite promising, as therapeutics targeting FcRn inhibition could provide a rapid and selective IgG lowering effect in a much less cumbersome method as compared to TPE. Thus, FcRn inhibitors could potentially be thought of as potential treatments for myasthenic crisis or as maintenance therapy.

Myasthenia gravis is an autoantibody-mediated disease with a favorable response to TPE treatment, so it is a prime disease for testing whether FcRn targeted treatments would be beneficial for antibody-mediated disease patient populations. Preclinical studies in the experimental animal model of MG support this treatment approach. A high affinity, pH-independent rat anti-FcRN inhibitor enhanced the clearance of pathogenic AChR antibodies and demonstrated a dose-dependent improvement in disease symptoms in both passive and active models of induced autoimmune MG. Therapeutic potential for FcRn agents was also demonstrated in a mouse model for muscle-specific kinase (MuSK) myasthenia gravis (16, 17). In addition to MG, this target for drug development is also being explored to expand treatment options for other autoimmune diseases, such as chronic inflammatory demyelinating polyneuropathy and idiopathic thrombocytopenic purpura.

## Safety Considerations for FcRn Targeted Therapeutics

Therapeutics targeting FcRn in clinical development for MG are human monoclonal antibodies or Fc fragments (Table 1). Given the specificity associated with these therapeutics, limited off-target effects are expected or have been observed in trials completed to date. In comparison to TPE, which removes many serum proteins, anti-FcRn therapeutics are expected to confer significant benefits in terms of fewer off-target effects. The current primary safety considerations with anti-FcRn therapies focus on the role of FcRn binding of albumin and the potential clinical implications of a reduction in serum albumin levels. Modest post-treatment reductions in albumin have been observed in preclinical studies and early phase studies in humans. However, to date there have been no demonstrable adverse clinical effects observed in the human clinical trials. In addition, severe depletion of IgG could theoretically increase the risk of infection. However, IgA, IgD, IgE, and IgM are not dependent on FcRn-mediated recycling and preliminary studies have not demonstrated any effect on the frequencies of immune cells (e.g., T, B cells, NK cells), complement, or peripheral cytokines (18). Furthermore, initiation of a primary immune response through IgM and IgG is expected to proceed intact (19). It is important to note that the long term effects of severe IgG depletion, particularly in the setting of additional immunosuppressive therapies as would be expected in many MG patients, remain uncertain and require additional study.

**Table 1:** Overview of FcRn targeted therapeutics in development for MG including Phase 1 trial results.

**Therapeutic Name**	**Company**	**Molecule Summary**	SAD/MAD Dosing^*^	**Maximum % IgG lowering from baseline in phase 1**
Efgartigimod (ARGX-113)	Argenx BVBA	Humanized IgG1 Fc fragment	**SAD** IV: 0.2, 2, 10, 25, 50 mg/kg	10, 25 mg/kg IV: 75%
			**MAD** **IV: 10**, 25 **mg/kg** every 4 or 7 days	
Nipocalimab (M281)	Momenta	Human deglycosylated IgG1 monoclonal Ab	**SAD** **IV:** 0.3, 3, 10, 30, **60 mg/kg**	15, 30 mg/kg IV: 85%
			**MAD** **IV:** 15, **30 mg/kg** weekly^†^	
Rozanolixizumab (UCB7665)	UCB Biopharma	Humanized IgG4 monoclonal Ab	**SAD** IV: 1, 4, 7 mg/kg **SC:** 1, **4, 7 mg/kg**	4 mg/kg SC: 26% 7 mg/kg SC: 43%
			**MAD** NA, SAD only	
RVT-1401(HL161)	Immunovant Sciences GmbH	Human IgG1 monoclonal Ab	**SAD** SC: 0.05, 1.5, 5 mg/kg, 340, 500, 765 mg IV: 1.5 mg/kg, 100 mg, 340, 765, 1,530 mg	340 mg SC: 63% 680 mg SC: 78%
			**MAD** **SC: 340, 680 mg** weekly	

*Ab, antibody; AChR, acetylcholine receptor; Ig, immunoglobulin; IV, intravenous; kg, kilogram; MAD, multiple ascending dose; mg, milligram; NA, not applicable; SAD, single ascending dose; SC, subcutaneous*.

* *Bolded doses used in MG phase 2 studies*.

† *A 5 mg/kg dose was also tested in phase 2*.

## FcRn Therapeutics in Development for MG

The remainder of the review focuses on four therapeutics currently in clinical testing for MG. One is a Fc fragment and three are anti-FcRn monoclonal antibodies. Table 1 summarizes each therapeutic and includes dosing and pharmacodynamic (PD) information from the phase 1 clinical trials that were critical to inform the subsequent clinical trials in MG patients. Pharmacokinetic (PK) parameters for each therapeutic may be found in the phase 1 and phase 2 manuscripts cited below. Other FcRn targeted strategies, such as recombinant Fc multimers and FcγR targeted therapeutics, and additional FcRn monoclonal antibodies are in development but have not yet entered clinical testing for MG (20, 21).

## Efgartigimod

This molecule is a modified human anti-IgG1 derived Fc fragment engineered to increase Fc/FcRn binding at neutral and acidic pH. Flow cytometry and microscopic data indicate a high affinity and avidity of efgartigimod for FcRn as evidenced by greater retention of efgartigimod in FcRn-positive compartments within cells, combined with increased lysosomal accumulation. Cynomolgus monkey studies demonstrated a maximal 75% reduction in IgG following multiple dosing up to 20 mg/kg and no significant safety concerns.

### Phase 1 Study

The safety, PK, and PD of efgartigimod was evaluated in a placebo-controlled single- and multiple ascending dose (SAD/MAD) study in 62 healthy adult volunteers (44 received efgartigimod) (21). In the SAD study, doses of 0.2–50 mg/kg administered as 2 h intravenous infusions were explored in 5 cohorts of healthy volunteers (Table 1). During the MAD portion of the study, 10 and 25 mg/kg doses administered every 4 or 7 days were studied. No dose-limiting toxicity was observed. Headache occurred in the highest dose of the SAD, was predominantly mild, and resolved with minimal interventions. There were no serious adverse events (SAEs) related to efgartigimod. A single dose reduced total IgGs about 50%, while repeated dosing lowered IgG levels by ~75% (21). The maximum IgG lowering effect was seen beginning with the 10 mg/kg dose and this dose was selected for further clinical development. There was no effect on other immunoglobulins or albumin at any of the studied doses. There was no significant anti-drug antibody production in the phase 1 study.

## Nipocalimab

This molecule is a human deglycosylated IgG1 anti-FcRn monoclonal antibody with no effector function. Nipocalimab binds with picomolar affinity to FcRn at both endosomal pH 6.0 and extracellular pH 7.6 allowing occupancy of FcRn throughout the recycling pathway and has a specificity designed to minimize off target effects (22). It is not expected to cross the placenta and a clinical trial is underway in pregnant women at high risk for early onset severe hemolytic disease of the fetus and newborn (23, 24). Phase 1 data supports infusion rates of 7.5 or 15 min for 30 and 60 mg/kg doses, respectively (25).

### Phase 1 Study

The phase 1 placebo-controlled study in healthy adult volunteers consisted of both SAD and MAD components and 50 subjects were enrolled (36 nipocalimab) (Table 1) (22). Doses of 0.3–60 mg/kg over a 2-h infusion were studied in the SAD cohorts, and the MAD cohorts included 4 weekly doses of 15 or 30 mg/kg. Greater than 90% FcRn receptor occupancy was achieved with ≥3 mg/kg doses within 2 h of dosing. Following single doses, maximum IgG reductions of 74–80% were observed with 30 or 60 mg/kg doses, and a ≥50% reduction in IgG levels were maintained for 18 and 27 days for the 30 or 60 mg/kg doses, respectively. During multiple doses, IgG levels were reduced ~85% below baseline by day 14. Treatment emergent AEs were similar in both the nipocalimab and placebo groups and most were mild or moderate. There were no severe or serious treatment emergent AEs, and there was no increase in the incidence of infections. Three subjects in the 15 mg/kg MAD experienced transient elevations in creatine phosphokinase and one of these cases was clearly related to exercise. Mild, transient reductions in total protein and albumin were observed in the highest SAD dose and in the MAD doses. The overall frequency of headache was similar between the nipocalimab and placebo groups in the trial.

## Rozanolixizumab

This molecule is a high affinity human anti-FcRn IgG4 monoclonal antibody. Rozanolixizumab dosing in animals demonstrated marked decreases in plasma IgG concentrations (75–90% from baseline) at 50 and 150 mg/kg doses with maximal effects achieved by about day 10. Rozanolixizumab does not strongly block albumin binding to FcRn, and small not clinically significant albumin decreases were observed in animals, possibly related to steric hindrance by antibody bound to FcRn. There was no increase in infection rates, no effects on plasma concentrations of acute-phase proteins, no changes in IgM and IgA serum concentrations and immunophenotyping did not show a significant treatment effect on absolute lymphocyte count or lymphocyte subsets (18).

### Phase 1 Study

Rozanolixizumab was studied in a placebo-controlled phase 1 trial in 49 healthy volunteer subjects (36 rozanolixizumab) administered as a single 1 h intravenous or subcutaneous infusion at doses of 1, 4, or 7 mg/kg (Table 1) (18). The intravenous formulation demonstrated a dose-dependent increase in headaches and back pain, including 4 severe treatment emergent AEs, compared to the subcutaneous formulation. Dose-dependent and treatment-related vomiting, nausea, and pyrexia were also seen more frequently in the intravenous formulation compared to placebo, and were less frequent with the subcutaneous formulation. As a result of these findings, subsequent clinical development has focused on the better tolerated subcutaneous formulation. The mean maximum IgG reduction following single doses of rozanolixizumab occurred at day 10 and was ~48% for the highest intravenous dose and 43% for the subcutaneous formulation.

## RVT-1401

### Phase 1 Study

Publically available preclinical and clinical trial data for RVT-1401 are more limited. RVT-1401 is a fully human monoclonal antibody formulated for intravenous or subcutaneous injection. A phase 1 SAD/MAD study in healthy volunteers has been completed (Table 1) (26). The SAD portion of the study included weight-based and fixed intravenous and subcutaneous doses (fixed doses 100–765 mg), while the MAD cohorts included administration of weekly subcutaneous doses of 340 or 680 mg RVT-1401 or placebo for 4 weeks. IgG levels were reduced by 47% after single doses of 765 mg, with the nadir being reached 8–10 days after dosing. Weekly subcutaneous dosing with 680 mg reduced total IgG levels by 78%. IgG reductions ≥35% were maintained for more than 1 month after the last dose. Reversible dose dependent reductions in albumin were observed (31% with 680 mg subcutaneous dosing) and were asymptomatic. Single and multiple doses of RVT-1401 were well-tolerated with no subjects terminating the study early due to AEs. The most common AEs in the phase 1 study were injection site erythema and swelling. No subjects in the MAD cohorts developed anti-drug antibodies.

## Clinical Trial Results in MG

The profound and rapid reductions in IgG concentrations and favorable PK and safety observed in the preclinical studies and healthy volunteer phase 1 studies supported further investigation anti-FcRn therapeutics in clinical trials in patients with MG. Of note, the rapid PD effects of the FcRn therapeutics confer the potential advantage of shorter duration clinical trials to demonstrate clinically meaningful results. Two therapeutics, efgartigimod and rozanolixizumab, have completed phase 2 clinical trials in patients with generalized MG and have active phase 3 programs. Phase 2 clinical trials for Nipocalimab and RVT-1401 are active. In summary, the phase 2 and phase 3 clinical trials studied adult patients with generalized MG. All trials included AChR antibody positive patients, while MuSK antibody positive patients were eligible for the nipocalimab and rozanolixizumab phase 2 trials. All these trials excluded patients with seronegative MG, with the exception of the efgartigimod phase 3 trial which included a limited number AChR- and MuSK antibody negative MG patients. Primary efficacy outcome measures included the MG-ADL and QMG score (Table 2). Several of the anti-FcRn clinical trial programs in MG include open-label extension studies.

**Table 2:** Summary of completed/active studies of anti-FcRn monoclonal antibodies for MG.

**Therapeutic name**	**Phase**	**Study design**	**Study population**	**Sample size**	**Dosing**	**Efficacy outcome measures and endpoints**	**Safety summary**	Results summary^**^
Efgartigimod (ARGX-113)	2	2-arm parallel RCT (1:1)	AChR-Ab	24	10 mg/kg efgartigimod or PBO weekly × 4 IV	Δ baseline to week 11: MGADL QMG MG-Composite MG-QoL15r	No SAE No severe TEAE One withdrawal due to lack of efficacy	**Efficacy week 11** MGADL: −3.5 QMG: −4.8 MG-Composite: −7.1 **Max PD** IgG: −71% AChR-Ab: −40–70%
Efgartigimod^*^ (ARGX-113)	3	2-arm parallel RCT (1:1)	AChR-Ab AChR-ab (includes MuSK-Ab, LRP4-Ab, Seronegative)^†^	150	10 mg/kg efgartigimod or PBO, dose adjustments allowed according to patient symptoms IV	Δ baseline to week 8:Primary:% MGADL responders in AChR-Ab pts Secondary:% QMG responders (AChR-Ab pts) % MGADL (all pts)	NA	NA
Nipocalimab^*^ (M281)	2	5-arm parallel RCT (1:1:1:1:1)	AChR-Ab MuSK-Ab	60	5 mg/kg M281 Q4 weeks, 30 mg/kg M281 Q4 weeks, 60 mg/kg M281 x1, 60 mg/kg M281Q2 week or PBO Q2 weeks × 4 IV	Δ baseline to week 8:Primary:MGADL Secondary:QMG MG-QoL15r	NA	NA
Rozanolixizumab (UCB7665)	2	Randomized controlled 2-period crossover study (1:1)^‡^	AChR-Ab MuSK-Ab	43	Period 1: 7 mg/kg rozanolixizumab or PBO weekly × 3 Period 2: 4 or 7 mg/kg rozanolixizumab weekly × 3 SC	Δ baseline to week 4:QMG MGADL MG-Composite	43.5% increase in TEAE of HA in active group; 3 active treatment subjects withdrew due to HA per protocol	**Efficacy week 4** QMG: −1.8 MGADL: −1.8 MG-Composite: −3.1 **Max PD** IgG Period 1: −56% IgG Period 2: −68% AChR-Ab Period 2: −68%
Rozanolixizumab^*^ (UCB7665)	3	3-arm parallel RCT (1:1:1)	AChR-Ab MuSK-Ab	240	7 mg/kg rozanolixizumab or PBO weekly × 3 SC	Δ baseline to week 6:Primary:MGADL Secondary: MG-composite QMG PRO fatigability	NA	NA
RVT-1401^*^	2	3-arm parallel RCT (1:1:1)	AChR-Ab	21	340 or 680 mg RVT-1401 or PBO Q2 weeks × 4 SC	Δ baseline to week 7:MGADL QMG MG-Composite MG-QoL15r	NA	NA

*Ab, antibody; AChR, acetylcholine receptor; ADL, activities of daily living; HA, headache; Ig, immunoglobulin; IV, intravenous; LRP4, low density lipoprotein receptor-related protein 4; MuSK, muscle-specific tyrosine kinase; NA, not available; PBO, placebo; PRO, patient-reported outcome; QMG, quantitative MG score; PD, pharmacodynamics QoL, quality of life; RCT, randomized controlled trial; SAE, serious adverse event; SC, subcutaneous; TEAE, treatment emergent adverse event*.

* *Study ongoing*.

** *Efficacy results should be interpreted with caution due to the different study designs and timing of primary efficacy endpoints assessments in the phase 2 trials*.

† *Limited to 30 patients*.

‡ *After period 1, subjects were re-randomized to 7 or 4 mg/kg rozanolixizumab*.

## Efgartigimod Clinical Development

### Phase 2 Study

The first data to directly support anti-FcRn therapy in human MG came through the phase 2 randomized, double-blind, placebo-controlled, multi-center clinical trial investigating efgartigimod in patients with AChR antibody positive generalized MG (27). Twenty-four patients were randomized 1:1 to placebo or 10 mg/kg efgartigimod intravenous infusions administered over 2 h on days 1, 8, 15, and 22. Patients were followed for 8 weeks after the last infusion.

Key eligibility criteria included a MG-ADL of at least 5 and a MGFA-severity class of II–IVa and stable immunosuppressive treatments. A history of malignancy and thymectomy within 3 months of screening were exclusionary. The primary endpoint was safety and secondary endpoints included change from baseline in validated MG clinical outcome measures, PK, and PD markers (Table 2).

Overall, efgartigimod was well-tolerated with no SAEs or treatment emergent AEs that led to discontinuation. No safety signals were identified. One patient with concomitant immunosuppressive drugs experienced a moderate AE of shingles. Maximum IgG level reductions were ~70% and AChR antibodies were reduced to a similar extent. AChR antibody levels returned to normal within 8 weeks of the last dose. Coinciding with maximal IgG lowering, improvements were observed in multiple MG outcome measures within 1–2 weeks of the last dose. The maximum change from baseline in MG outcome measures were: QMG −5.7 points, MG-ADL −4.4, MG-Composite −9.4, and MG-QOL15r −6.0. Reductions in the QMG and MG-ADL were statistically significant at day 8 (QMG) and days 29 and 36 (MG-ADL) (25). Efficacy endpoint reductions at the end of the study are shown in Table 2. In addition, 75% of patients who received efgartigimod had a ≥2 point improvement in the MG-ADL score for ≥6 weeks, whereas only 25% of placebo patients experienced a similar effect (28).

Given these promising results, a phase 3 randomized, double-blind, placebo-controlled, multi-center clinical trial is currently ongoing with a primary endpoint assessing the percentage of MG-ADL responders at 8 weeks among AChR antibody positive patients (29).

## Nipocalimab Clinical Development

### Phase 2 Study

In the phase 2, randomized, double-blind, placebo-controlled study, 60 patients with generalized AChR- or MuSK antibody positive MG are planned to be enrolled in 4 active and 1 placebo treatment arms (Table 2) (30). Each participant will receive a total of 5 study intravenous infusions administered every 2 weeks. The primary endpoints are safety and the change from baseline in the MG-ADL score at day 57. Secondary outcome measures include the change from baseline in the QMG and MG-QOL15r scores, as well as the change in serum IgG levels. The active treatment arms will evaluate multiple dosing regimens thereby providing PD data in patients that should help optimize dosing for this therapeutic in patients.

## Rozanolixizumab Clinical Development

### Phase 2 Study

UCB Pharma has completed a phase 2, randomized, double-blind, placebo-controlled, clinical trial in patients with generalized MG. Forty-three patients were randomized to three once per week subcutaneous infusions of placebo or 7 mg/Kg rozanolixizumab on days 1, 8, and 15 (Period 1). Patients were followed for 4 weeks after the last infusion and then were re-randomized to 3 doses of either 4 or 7 mg/kg rozanolixizumab (Period 2). Standard of care MG treatments were kept stable during the study.

Key eligibility criteria included adult patients with AChR- and MuSK antibody positive generalized MG patients who could be considered for IVIg or PLEX treatment in the opinion of the investigator and who had a QMG score of at least 11 (31).

Similar to the efgartigimod phase 2 trial, the primary outcome was safety. Of note, 57% of patients treated with rozanolixizumab experienced headache and three patients were withdrawn from the study due to headache (32). There was no difference in the rate of infections between the active and placebo treatment groups. At the end of Period 1, there was a statistically significant, but marginally clinically significant, improvement in the change from baseline MG-ADL score in the rozanolixizumab group. The MG-ADL responder rate, defined as a reduction of three or more points from baseline, more robustly favored rozanolixizumab (47.6 vs. 13.6% for placebo). Other MG outcome measures were not significant. During Period 2 further improvements were observed in the rozanolixizumab group, where the high dose group experienced improvements of −5.1, −8.5, and −3.9 points on the QMG, MG-Composite, and MG-ADL scores, respectively. The phase 2 trial demonstrated a 68% decrease in serum IgG and AChR autoantibodies at the end of Period 2 (33).

A 240 patient, phase 3, parallel design, randomized, double-blind, placebo-controlled, multi-center clinical study of rozanolixizumab is currently ongoing (34). Patients will be randomized to one of two rozanolixizumab doses or placebo. The primary endpoint is the MG-ADL score change from baseline at day 43 among AChR antibody positive patients (Table 2).

## RVT-1401 Clinical Development

### Phase 2 Study

The phase 2 parallel group clinical trial will evaluate the safety and PD effects of subcutaneous RVT-1401 in 21 adult AChR antibody MG patients (35). The two active drug arms and a placebo arm will treat patients for 6 weeks. Patients must have a QMG score ≥12 prior to randomization. Efficacy endpoints include the MG-ADL, QMG, and MG-Composite (Table 2).

## Clinical Development Summary

The available phase 1 and phase 2 clinical trial data for anti-FcRn monoclonal antibodies consistently demonstrate the ability to reduce and maintain total IgG and/or AChR autoantibodies at levels associated with efficacy for PLEX (5, 36). In general, all of the available therapeutics reduce IgG levels by 60–80% from baseline at the doses studied, with modest effects on albumin. As expected, all of the therapeutics show a selective effect on IgG with no significant changes in IgA, IgD, IgE, and IgM.

At the moment, differences in therapeutic administration, such as the route and infusion duration, and side effects are the primary clinical features distinguishing these therapeutics in the early phase clinical trials. All were generally well-tolerated in phase 1 studies with headaches from rozanolixizumab being the most potentially limiting AE identified to date. In the short term studies to date, no serious infections were observed. The ongoing phase 2 and phase 3 programs will undoubtedly add critical information to our understanding of these therapies and their distinguishing features.

## Discussion and Conclusions

Preliminary results of the completed rozanolixizumab and efgartigimod phase 2 trials suggest proof of concept for IgG lowering strategies to treat MG. The ongoing phase 2 and phase 3 trials will provide additional needed efficacy and safety data, though the long term safety profile of >70% reductions in total plasma IgG levels will not be available in the near term. Of particular interest is the risk of hypogammaglobulinemia associated infections in patients with MG who are typically receiving chronic immunosuppressive agents. Determination of dosing and the degree of IgG lowering needed for chronic therapy are essential and the nipocalimab phase 2 program, which has four active dosing arms, should provide important data in that regard. Immunogenicity is also uncertain, but preliminary results suggest low immunogenic potential for all of the therapeutics in development for MG.

If the efficacy and safety of anti-FcRn therapeutics are confirmed in pivotal trials, this class of therapy may be able to address limitations of the existing rapidly acting treatments, plasma derived immunoglobulins and TPE, which include limited supply/availability (IVIg), prolonged treatment durations (TPE/IVIg), large infusion volumes (IVIg), and adverse effects. In addition, clinicians will be very interested in comparative trial data for FcRn targeted therapeutics and TPE/IVIg for the inpatient treatment of MG, which is the setting where IgG lowering is presently used most commonly and which has not been studied to date. Multiple ongoing clinical trials with FcRn antibodies and complement therapeutics in MG patients have predictably established that there are a limited number of patients available for traditional clinical trials (37). How this competition for clinical sites and eligible patients will play out in future clinical trials remains to be seen. When viewed from the broader context of emerging therapeutics for MG, targeted combination therapies with distinct and complementary mechanisms, such as FcRn targeted therapies in combination with complement therapeutics, should be studied to determine whether they provide additional efficacy with favorable safety over existing regimens.

## Author Contributions

JG and KG wrote and revised the manuscript.

### Conflict of Interest

JG has served as a consultant for companies developing therapies described in the manuscript, served as an investigator for FcRn clinical trials in MG sponsored by Momenta and UCB, and served as DSMB member for Argenx. The remaining author declares that the research was conducted in the absence of any commercial or financial relationships that could be construed as a potential conflict of interest. The handling Editor declared a past co-authorship with one of the authors JG.
